# Willingness of population health survey participants to provide personal health information and biological samples

**DOI:** 10.1186/s12889-024-20769-2

**Published:** 2024-11-26

**Authors:** Harpreet Jaswal, Anca Ialomiteanu, Hayley Hamilton, Jürgen Rehm, Samantha Wells, Kevin D. Shield

**Affiliations:** 1 Institute for Mental Health Policy Research, Centre for Addiction and Mental Health, 33 Ursula Franklin Street, Toronto, ON M5S 2S1 Canada; 2 Dalla Lana School of Public Health, University of Toronto, 155 College St, Toronto, ON M5T 3M7 Canada; 3 Campbell Family Mental Health Research Institute, Centre for Addiction and Mental Health, 33 Ursula Franklin Street, Toronto, ON M5S 2S1 Canada

**Keywords:** Survey, Data linkage, Biological sample, Substance use, Mental health

## Abstract

**Background:**

Biological sample collection and data linkage can expand the utility of population health surveys. The present study investigates factors associated with population health survey respondents’ willingness to provide biological samples and personal health information.

**Methods:**

Using data from the 2019 Centre for Addiction and Mental Health (CAMH) Monitor survey (*n* = 2,827), we examined participants’ willingness to provide blood samples, saliva samples, probabilistic linkage, and direct linkage with personal health information. Associations of willingness to provide such information with socio-demographic, substance use, and mental health details were also examined. Question order effects were tested using a randomized trial.

**Results:**

The proportion of respondents willing to provide blood samples, saliva samples, probabilistic linkage, and direct linkage with personal health information were 19.9%, 36.2%, 82.1%, and 17%, respectively. Willingness significantly varied by age, race, employment, non-medical prescription opioid use (past year), cocaine use (lifetime), and psychological distress. Significant question order effects were observed. Respondents were more likely to be willing to provide a saliva sample when this question was asked first compared to first being asked for direct data linkage. Similarly, respondents were more likely to be willing to allow for probabilistic data linkage when this question was asked first compared to first being asked for a saliva sample.

**Conclusion:**

A lack of willingness to provide biological samples or permit data linkage may lead to representivity issues in studies which rely on such information. The presence of question order effects suggests that the willingness of respondents can be increased through strategic ordering of survey structures.

**Supplementary Information:**

The online version contains supplementary material available at 10.1186/s12889-024-20769-2.

## Introduction

Population health surveys are the cornerstone of population health surveillance by providing population estimates of the prevalence and patterns of health conditions and risk factors [1, 2]. However, numerous population health surveys utilize cross-sectional designs, thus making it difficult to ascertain causality. Furthermore, retrospective assessments and self-reported data are inherently subject to reliability issues due to their reliance on the recall of information and events, and on individuals’ propensity to report [3].

Data linkage to personal health information can be employed to overcome these limitations of cross-sectional population surveys [4]. Personal health information (PHI) is any identifying information about an individual related to their mental or physical health, family history, or health care history [5]. In Ontario, most of an individual’s PHI is centralized within their Ontario Health Insurance Plan (OHIP). Examples of PHI include dates and types of health services received, laboratory testing, and health-related claims [6]. This repository of health information can be linked to population surveys through an individual’s unique OHIP number.

Linking population surveys to PHI expands the utility of valuable, existing data that were not originally collected for research purposes [4]. Such linking enables researchers to derive aggregate health trends in populations that can be used for policy development. Furthermore, linking PHI and population surveys can be leveraged to build longitudinal health profiles of patients, which in turn can inform the monitoring, evaluation, and delivery of health care [7, 8].

Population surveys can be further improved through the collection of biological samples [9, 10]. The collection of biological samples, such as blood, feces, urine, and saliva, alongside survey administration data, circumvents the time and costs associated with primary data collection of biological samples in follow-up studies, and curates a more comprehensive and larger data set [10, 11]. Large sample sizes are needed for the detection of genetic markers, and for reliable and valid outcome information. Within the context of substance use, biological samples can be used to yield insight into the genotypic underpinnings of observable behaviours and traits associated with substance use and substance use disorders [12]. The National Epidemiologic Survey on Alcohol and Related Conditions-III (NESARC-III) employs a cross-sectional design to capture information on alcohol and drug use disorders and related factors, alongside the collection of saliva samples for the assessment of genetic factors [13]. Such information is collected to promote new scientific knowledge regarding the role of genetics and environmental factors in the initiation and endurance of substance use disorders and their associated disabilities [14]. Similarly, when linked to questionnaires and record-based mental health data, salivary genetic samples can be used to identify and understand the risks and protective factors associated with psychiatric conditions (e.g., anxiety and other mood-related disorders) and the development of dependence on psychoactive substances [15, 16]. Similarly, the linkage of survey data to health records can aid in our understanding of the service utilization and health needs of people with psychiatric conditions (e.g., anxiety and other mood-related disorders) and of people who are dependent on psychoactive substances. Currently, to our knowledge, there are few to no population health surveys in Canada that systematically collect biological samples for data linkage [17].

There are a number of identified issues that hinder the linkage of PHI to population health surveys and the collection of biological samples. Though study participants simultaneously assume the roles of primary beneficiaries and risk bearers in sharing personal information for linkage with survey data, their perspectives on these benefits and risks are not well characterized [18]. In a previous study, some participants expressed concern over the risk of PHI security being breached, the adequacy of the de-identification of data, and their information being disclosed to employers [19]. Furthermore, patients’ reluctance towards sharing personal information is often influenced by a perceived or real lack of transparency about how their data are being used.

Willingness to provide biological samples and permission for linkage may be associated with respondents’ demographic characteristics. It has been frequently observed that willingness to participate in health research and provide health information varies by ethnicity, age, and socio-economic and martial status [20–22]. For example, people who are younger, married, and/or highly educated are frequently found to have increased willingness to engage in such research [20–22]. Studies examining differences by sex in the willingness to participate in health research have provided mixed results; some studies have found that males are more likely to participate in such research, and other studies have found no differences by sex in the willingness to participate [20–22].

Lower levels of participation by specific socio-demographic groups have been previously attributed to various health system issues and discriminatory events within research practice [23]. The frequent underrepresentation of these groups in health studies can contribute to health disparities alongside negative consequences for equity, ethics, and scientific rigor—thus necessitating the investigation of socio-demographic characteristics in relation to willingness to provide biological samples and PHI [24].

Participants’ willingness to provide PHI could also be affected by the ordering of the questions in the survey. Order effects bias is a type of response bias in which the sequence of two or more questions pertaining to similar issues can influence the answers obtained [25]. In other words, the order in which questions are asked could increase or decrease a participant’s propensity to share their PHI for data linkage. Currently, to our knowledge, there are no studies that directly examine whether participants’ responses differ depending upon the order of questions regarding willingness to provide PHI information or biological samples.

To address these highlighted gaps in the research, the objectives of the current study were to:

1. estimate the proportion of Ontario adults who were willing to provide biological samples and PHI for linkage to their survey responses;
2. examine associations of willingness to provide such information with (i) demographic variables, including gender, age, race, marital status, education, employment status, and rurality, (ii) substance use variables, including harmful/hazardous alcohol use, cannabis use, cocaine use, and non-medical prescription opioid use (NMPOU), and (iii) mental health status; and.
3. assess, using a randomized trial, whether the timing of when PHI and biological samples were requested affected respondents’ agreement to provide such information for data linkage.

## Methods

The current study was based on data derived from the 2019 cycle of the CAMH Monitor survey. With the use of survey weights to recover population representation, this cycle of the survey is considered to be representative of 10,766,695 Ontarians aged 18 and older, from English-speaking households. To increase the response rate in Ontario, all households selected to be telephoned were mailed a letter informing them of the study details and that they would be contacted by phone in the near future. Participants were then contacted and surveyed via telephone interviews. Full details on the methodology utilized can be found in the 2019 CAMH Monitor eReport [26].

### Measures

The main measures of interest included the following: willingness to provide a saliva sample, willingness to provide a blood sample, willingness to have survey data linked through probabilistic linkage, and willingness to have survey data linked through direct linkage (i.e., providing an OHIP card number). Willingness to provide a saliva sample and a blood sample was assessed by asking participants if they “would … be willing to provide a saliva sample to University of Toronto and CAMH researchers” and if they “would you be willing to provide a blood dot sample to University of Toronto and CAMH researchers”.

Probabilistic linkage is defined as the matching of survey responses to the Institute for Clinical and Evaluative Sciences (ICES) databases. The process of probabilistic linkage was conveyed to survey participants by informing them that “researchers from CAMH and University of Toronto would like to share your responses from this interview with researchers from the Institute for Clinical and Evaluative Sciences, a non-profit research organization funded by the Ontario Ministry of Health and Long Term Care, so researchers can evaluate and improve Ontario’s health care system”. Participants were then asked if they were “willing to give permission to do this”.

Direct linkage occurs through the provision of the respondent’s OHIP number for the linkage of survey responses to the ICES databases. The process of direct linkage was conveyed to survey participants by informing them that “researchers from CAMH and University of Toronto would like to link your responses from this interview to existing provincial health information. This information would be used by CAMH and University of Toronto researchers to evaluate and improve Ontario’s health care system. In order to link your survey responses to existing provincial health information, we would need your OHIP number.” Respondents were then asked to “provide the 10 digit number and 2 letter version code located on the front of [their] OHIP card”.

The binary-response answers to these questions (i.e., yes / no, refused) (with the addition of the OHIP number for direct data linkage) were used to assess participants’ willingness to provide biological sample and to have their PHI linked to a public health insurance database. The complete wording of the questions used to measure a participant’s willingness to provide biological samples and to have their PHI linked to a public health insurance database is outlined in the supplement.

The main explanatory socio-demographic variables of interest were gender, age, race, marital status, education, employment, rurality, all of which have been previously observed to be associated with willingness to participate in medical research studies [20–22]. The main substance use/misuse explanatory variables were Alcohol Use Disorders Identification Test (AUDIT) scores, frequency of cannabis use in the past 12 months (never, monthly to weekly, daily), non-medical prescription opioid use (NMPOU) in the past 12 months (yes/no), lifetime cocaine use (yes/no). The AUDIT is a screening tools used to measure early stage alcohol problems and the Kessler Psychological Distress Scale (K6) is a measure of level of psychological distress [27, 28]. As per the World Health Organization (WHO) guidelines, an AUDIT score was categorized using 1 to 7 to indicate low-risk consumption, while scores greater than 7 were used to indicate moderate to high risk consumption [29]. The main psychometric explanatory variable was the K6 score [27, 28]. The K6 variable was categorized using the cut-off score of 8 or higher which indicates ‘moderate to serious’ pscychological distress [30].

## Randomized trial

A randomized trial was employed with two levels of randomization producing eight randomized groupings. The first level of randomization was to determine what group of questions was to be asked first (the request for a biological sample or the request for PHI). The second level of randomization was to determine, firstly, what biological sample was to be requested: [1] a blood sample, or [2] a saliva sample, and, secondly, what PHI was to be requested: [1] permission to link (probabilistic) based on the last name, birth year, and postal code of the respondent, or [2] a direct linkage request using a participant’s OHIP number. Randomization was performed using a pre-generated unbalanced randomized sequence.

## Analysis

A series of multivariate quasi-Poisson general linear regression models, which accounted for survey design and weights, were used to compare the willingness of participants to provide biological samples and PHI by the selected predictor variables. Quasi-Poisson general linear regression models produce prevalence ratios when binary outcome data are modelled [31]. Multivariate quasi-Poisson general linear regression models were used to analyze the data from the randomized trial to see if question order affected the willingness of respondents to provide biological samples and PHI. Differences in demographic variables by randomized group were assessed by means of chi-squared tests.

Survey weights were used to restore population representation, as the CAMH Monitor survey sample was equally allocated within each of the six regional strata. The survey weights used in this analysis were a function of the inclusion probability and a post-stratification adjustment. All statistics were performed using the statistical software package R version 1.4.1106, and the R statistical package “survey” [32].

## Results

The 2019 CAMH Monitor survey response rate was 28% (*n* = 2827 interviews) from January 2, 2019 to December 19, 2019. Willingness to provide a blood sample and willingness to provide a saliva sample had valid data for *n* = 412 (effective response rate of 26%) and *n* = 428 (effective response rate of 26%), respectively. Probabilistic linkage with the health insurance database had valid data for *n* = 405 (effective response rate of 26%), while direct linkage with the health insurance database had valid data for *n* = 435 (effective response rate of 26%). Unweighted sample sizes are listed in Tables S1 and S2 in the supplement.

Tables 1 and 2 depict the sample characteristics by the following study groups: blood sample, saliva sample, probabilistic linkage with a health insurance database, and direct linkage with a health insurance database (using a health insurance number). Small proportions of respondents were willing to provide blood samples (19.9%, 95% CI: 15.3–24.5), saliva samples (36.2%, 95% CI: 30.7–41.7), and have their survey responses directly linked with the health insurance database (17%, 95% CI: 12.6–21.4). In contrast, many respondents were willing to have their PHI probabilistically linked (82.1%, 95% CI: 77.5–86.7). Across all study groups, weighted prevalence estimates were higher for people who were White, married, employed, low-risk drinkers, living in non-rural areas, cannabis abstainers, NMPOU abstainers, cocaine abstainers, and those who had K6 scores below 8.

**Table 1:** Prevalence of willingness for biological sample collection by demographic, substance use, and mental health variables, by study group, 2019 CAMH Monitor study

Demographics		Study group						
		Blood sample				Saliva sample		
		Prevalence (%)	95% CIs	Sample size		Prevalence (%)	95% CIs	Sample size
Agreed to provide sample	Yes	19.9	(15.3, 24.5)	94		36.2	(30.7, 41.7)	169
	No	80.1	(75.5, 84.7)	318		63.8	(58.3, 69.3)	259
Gender	Men	46.0	(39.9, 52.1)	167		51.2	(45.4, 56.9)	187
	Women	54.0	(47.9, 60.1)	245		48.8	(43.1, 54.6)	241
Age (years of age)	18–29	22.6	(17.2, 28.1)	68		21.6	(16.4, 26.8)	70
	30–39	14.0	(9.6, 18.3)	41		14.4	(10.0, 18.8)	42
	40–49	13.5	(9.3, 17.8)	50		15.1	(10.4, 19.8)	48
	50–64	28.4	(22.9, 34.0)	101		27.8	(22.7, 32.9)	117
	65–74	11.8	(8.6, 15.0)	80		12.7	(9.4, 15.9)	83
	*≥* 75	9.7	(7.1, 12.2)	72		8.4	(6.1, 10.7)	68
Race	White	79.4	(74.5, 84.4)	345		74.8	(69.4, 80.3)	354
	Other	20.6	(15.6, 25.5)	67		25.2	(19.7, 30.6)	74
Marital status	Married	54.9	(48.9, 60.9)	224		60.0	(54.2, 65.7)	238
	Widowed	6.6	(4.5, 8.6)	58		4.3	(2.9, 5.8)	45
	Divorced / separated	6.8	(3.8, 9.8)	30		8.9	(5.9, 11.8)	49
	Never married	31.7	(25.8, 37.7)	100		26.8	(21.4, 32.3)	96
Education	University degree	33.7	(28.1, 39.4)	133		34.4	(28.6, 40.2)	132
	College diploma	32.2	(26.5, 37.9)	123		28.9	(23.5, 34.3)	119
	Completed high school	27.5	(22.1, 32.8)	121		30.1	(25.0, 35.3)	140
	Did not graduate high school	6.6	(3.9, 9.3)	35		6.6	(4.2, 9.0)	37
Employment	Employed / student / disability / homemaker	71.0	(65.9, 76.1)	248		72.1	(67.3, 76.8)	255
	Unemployed	3.2	(1.0, 5.4)	11		2.7	(0.8, 4.5)	9
	Retired	23.8	(19.3, 28.4)	145		24.5	(20.1, 28.9)	161
	Other	1.9	(0.2, 3.6)	8		0.8	(0.0, 1.7)	3
Rural	No	85.0	(81.2, 88.9)	335		81.3	(77.3, 85.4)	340
	Yes	15.0	(11.1, 18.8)	77		18.7	(14.6, 22.7)	88
AUDIT score	0 (abstain)	24.7	(19.5, 29.9)	102		19.1	(14.7, 23.4)	89
	1 to 7 (low risk)	63.0	(57.1, 68.8)	267		69.0	(63.7, 74.4)	293
	> 7 (moderate to high risk)	12.3	(8.3, 16.3)	43		11.9	(8.0, 15.8)	46
Cannabis use frequency	Never	79.8	(74.8, 84.8)	342		73.4	(68.0, 78.9)	333
	Monthly to weekly	15.0	(10.5, 19.6)	51		20.7	(15.6, 25.9)	72
	Daily	5.2	(2.6, 7.7)	19		5.8	(3.2, 8.4)	23
Non-medical prescription opioid use	No	94.6	(92.1, 97.1)	391		96.8	(94.7, 98.8)	410
	Yes	5.4	(2.9, 7.9)	21		3.2	(1.2, 5.3)	16
Cocaine use (lifetime)	No	92.6	(89.5, 95.7)	384		89.3	(85.4, 93.1)	393
	Yes	7.4	(4.3, 10.5)	28		10.7	(6.9, 14.6)	35
K6 score	0 to 7	80.5	(75.6, 85.4)	346		77.6	(72.4, 82.8)	354
	≥8	19.5	(14.6, 24.4)	66		22.4	(17.2, 27.6)	74

**Table 2:** Weighted sample demographics for the probabilistic and direct data linkage study groups in the 2019 CAMH Monitor study

Demographics		Study group						
		Probabilistic linkage with a health insurance database				Direct linkage with a health insurance database (using a health insurance number)		
		Prevalence (%)	95% CIs	Sample size		Prevalence (%)	95% CIs	Sample size
Agreed to provide data	Yes	82.1	(77.5, 86.7)	335		17.0	(12.6, 21.4)	79
	No	17.9	(13.3, 22.5)	70		83.0	(78.6, 87.4)	356
Gender	Men	44.4	(38.3, 50.5)	161		52.3	(46.5, 58.1)	193
	Women	55.6	(49.5, 61.7)	244		47.7	(41.9, 53.5)	242
Age (years)	18–29	24.9	(19.3, 30.5)	75		19.7	(14.6, 24.8)	63
	30–39	13.2	(8.9, 17.4)	40		15.0	(10.6, 19.5)	43
	40–49	10.6	(6.6, 14.6)	36		17.5	(12.8, 22.3)	62
	50–64	29.5	(23.9, 35.1)	106		26.9	(21.8, 32.0)	112
	65–74	13.2	(9.8, 16.6)	85		11.5	(8.4, 14.6)	78
	≥75	8.6	(6.3, 11.0)	63		9.3	(6.9, 11.8)	77
Race	White	79.5	(74.3, 84.8)	339		74.9	(69.7, 80.1)	360
	Other	20.5	(15.2, 25.7)	66		25.1	(19.9, 30.3)	75
Marital status	Married	57.3	(51.3, 63.3)	222		57.7	(51.9, 63.5)	240
	Widowed	5.1	(3.3, 6.9)	46		5.6	(3.9, 7.3)	57
	Divorced / separated	8.3	(5.2, 11.4)	41		7.5	(4.6, 10.4)	38
	Never married	29.3	(23.5, 35.1)	96		29.1	(23.5, 34.7)	100
Education	University degree	33.8	(27.9, 39.6)	123		34.3	(28.7, 39.9)	142
	College diploma	30.0	(24.4, 35.5)	115		30.9	(25.4, 36.4)	127
	Completed high school	29.1	(23.8, 34.5)	129		28.6	(23.4, 33.8)	132
	Did not graduate high school	7.1	(4.6, 9.6)	38		6.2	(3.6, 8.8)	34
Employment	Employed / student / disability / homemaker	70.7	(65.5, 75.8)	244		72.3	(67.6, 77.0)	259
	Unemployed	2.3	(0.4, 4.3)	7		3.5	(1.4, 5.5)	13
	Retired	26.2	(21.3, 31.0)	149		22.5	(18.4, 26.6)	157
	Other	0.8	(0.0, 1.7)	5		1.7	(0.1, 3.4)	6
Rural	No	83.9	(80.0, 87.9)	330		82.4	(78.4, 86.4)	345
	Yes	16.1	(12.1, 20.0)	75		17.6	(13.6, 21.6)	90
AUDIT score	0 (abstain)	21.1	(16.2, 26.0)	92		22.4	(17.7, 27.1)	99
	1 to 7 (low risk)	68.9	(63.3, 74.5)	273		63.7	(58.1, 69.3)	287
	> 7 (moderate to high risk)	10.0	(6.4, 13.7)	40		13.9	(9.7, 18.1)	49
Cannabis use frequency	Never	74.5	(68.8, 80.2)	324		78.2	(73.3, 83.1)	351
	Monthly to weekly	21.5	(16.0, 27.0)	64		15.0	(10.7, 19.4)	59
	Daily	4.0	(1.9, 6.1)	17		6.8	(3.9, 9.6)	25
Non-medical prescription opioid use	No	95.9	(93.4, 98.4)	392		95.5	(93.5, 97.6)	409
	Yes	4.1	(1.6, 6.6)	13		4.5	(2.4, 6.5)	24
Cocaine use (lifetime)	No	91.3	(87.7, 94.9)	378		90.5	(87.0, 94.0)	399
	Yes	8.7	(5.1, 12.3)	27		9.5	(6.0, 13.0)	36
K6 score	0 to 7	80.7	(75.7, 85.8)	341		77.5	(72.5, 82.6)	359
	≥8	19.3	(14.2, 24.3)	64		22.5	(17.4, 27.5)	76

Table 3 presents the associations between respondents’ willingness to provide biological samples and the aforementioned demographic, substance use, and mental health variables. Prevalence ratios (PR) of 1 are interpreted as an increased willingness to provide biological samples or PHI for data linkage relative to the reference group, whereas PRs less than 1 are interpreted as a decreased willingness [33]. Gender, race, employment status, rurality, AUDIT score, cannabis use frequency, and NMPOU were not significantly associated with willingness to provide a blood sample. People 75 years of age and older were more willing to provide a blood sample than people 30–39 years of age (PR: 4.24, 95% CI: 1.34–13.36). Similarly, being divorced or separated (PR: 2.04, 95% CI: 1.23–3.37), a lifetime cocaine user (PR: 3.18, 95% CI: 1.36–7.41), and having a K6 score equal to or greater than 8 (1.77, 95% CI: 1.07–2.91) were significantly associated within an increased willingness to provide a blood sample. Respondents who completed high school were less willing to provide blood samples than those who had completed a university degree (PR: 0.51, 95% CI: 0.28–0.92). Age, marital status, education, employment, rurality, AUDIT score, cannabis use in the last 12 months, NMPOU, lifetime cocaine use, and K6 scores were not significantly associated with willingness to provide a saliva sample. Respondents who reported their race as ‘Other’ were less willing to provide a saliva sample than were White respondents (PR: 0.56, 95% CI: 0.32–0.99).

**Table 3:** Prevalence ratios of willingness for biological sample collection by demographic, mental health, and substance use variables, 2019 CAMH Monitor study

Demographics		Blood sample				Saliva sample		
		Prevalence ratio	95% CIs	*p*-value		Prevalence ratio	95% CIs	*p*-value
Gender	Women	0.98	(0.64, 1.49)	0.922		0.93	(0.67, 1.28)	0.646
	Men	REF	-	-		REF	-	-
Age (years)	18–29	2.19	(0.54, 8.98)	0.275		0.61	(0.31, 1.19)	0.145
	30–39	REF	-	-		REF	-	-
	40–49	2.47	(0.87, 7.06)	0.091		0.64	(0.31, 1.32)	0.224
	50–64	1.92	(0.76, 4.83)	0.169		0.86	(0.53, 1.40)	0.548
	65–74	1.93	(0.68, 5.46)	0.214		0.73	(0.38, 1.38)	0.333
	≥75	4.24	(1.34, 13.36)	0.014		0.83	(0.42, 1.65)	0.597
Race	White	REF	-	-		REF	-	-
	Other	0.62	(0.30, 1.27)	0.196		0.56	(0.32, 0.99)	0.049
Marital status	Married	REF	-	-		REF	-	-
	Widowed	0.68	(0.36, 1.31)	0.252		0.99	(0.63, 1.56)	0.971
	Divorced / separated	2.04	(1.23, 3.37)	0.006		1.13	(0.72, 1.79)	0.589
	Never married	0.49	(0.18, 1.31)	0.156		0.81	(0.49, 1.34)	0.414
Education	University degree	REF	-	-		REF	-	-
	College diploma	0.58	(0.32, 1.04)	0.069		0.74	(0.49, 1.10)	0.137
	Completed high school	0.51	(0.28, 0.92)	0.025		1.00	(0.69, 1.45)	0.990
	Did not graduate high school	0.48	(0.23, 1.00)	0.051		0.99	(0.59, 1.67)	0.981
Employment	Employed / student / disability / homemaker	REF	-	-		REF	-	-
	Unemployed	1.22	(0.28, 5.25)	0.789		1.14	(0.40, 3.27)	0.804
	Retired	1.00	(0.54, 1.85)	0.999		1.07	(0.68, 1.69)	0.773
	Other	1.79	(0.55, 5.87)	0.335		0.47	(0.06, 3.66)	0.472
Rural	No	REF	-	-		REF	-	-
	Yes	1.48	(0.89, 2.47)	0.132		1.18	(0.87, 1.60)	0.296
AUDIT score	0 (abstain)	REF	-	-		REF	-	-
	1 to 7 (low risk)	0.96	(0.58, 1.59)	0.879		0.97	(0.68, 1.40)	0.886
	> 7 (moderate to high risk)	0.44	(0.16, 1.21)	0.112		1.01	(0.58, 1.75)	0.977
Cannabis use frequency	Never	REF	-	-		REF	-	-
	Monthly to weekly	1.52	(0.84, 2.77)	0.169		1.03	(0.63, 1.67)	0.913
	Daily	0.62	(0.16, 2.50)	0.506		0.99	(0.55, 1.81)	0.987
Non-medical prescription opioid use	No	REF	-	-		REF	-	-
	Yes	1.68	(0.88, 3.24)	0.119		0.79	(0.31, 1.98)	0.614
Cocaine use (lifetime)	No	REF	-	-		REF	-	-
	Yes	3.18	(1.36, 7.41)	0.008		0.63	(0.35, 1.15)	0.132
K6 score	0 to 7	REF	-	-		REF	-	-
	≥8	1.77	(1.07, 2.91)	0.026		1.24	(0.86, 1.80)	0.254

Table 4 presents respondents’ willingness to provide PHI by the aforementioned variables. NMPOU (PR: 1.45, 95% CI: 1.19–1.78) and being of an ‘Other’ race (PR: 0.81, 95% CI: 0.66–0.99) were significantly associated with willingness for probabilistic linkage with the health insurance database. Gender, age, marital status, education, employment, rurality, AUDIT score, cannabis use in the past 12 months, cocaine use (lifetime), and K6 score were not significantly associated with probabilistic linkage with the health insurance database. People who did not graduate high school had a significantly lower willingness to permit direct data linkage than those with a university degree (PR: 0.33, 95% CI: 0.15–0.76). People who engaged in NMPOU were less willing to provide direct linkage with the health insurance database than were people who did not engage in NMPOU (PR: 0.18, 95 CI: 0.03–0.97). People who had used cocaine in their lifetime had a higher willingness to provide direct linkage than those who had not used cocaine in their lifetime (95% CI: 1.37, 6.72). Gender, race, marital status, employment, rurality, AUDIT score, cannabis use, and K6 score were not significantly associated with willingness to provide direct linkage with the health insurance database.

**Table 4:** Prevalence ratios of willingness for probabilistic and direct data linkage by demographic, mental health, and substance use variables, 2019 CAMH Monitor study

Demographics		Probabilistic linkage with the health insurance database				Direct linkage with the health insurance database (using a health insurance number)		
		Prevalence ratio	95% CIs	*p*-value		Prevalence ratio	95% CIs	*p*-value
Gender	Women	0.94	(0.84, 1.04)	0.229		0.70	(0.44, 1.14)	0.153
	Men	REF	-	-		REF	-	-
Age (years)	18–29	0.83	(0.68, 1.03)	0.094		1.34	(0.39, 4.59)	0.647
	30–39	REF	-	-		REF	-	-
	40–49	0.91	(0.73, 1.14)	0.410		2.39	(0.72, 7.86)	0.153
	50–64	1.01	(0.84, 1.21)	0.932		1.76	(0.60, 5.12)	0.302
	65–74	0.87	(0.67, 1.12)	0.271		2.07	(0.58, 7.36)	0.261
	≥75	0.91	(0.68, 1.20)	0.487		2.31	(0.62, 8.61)	0.211
Race	White	REF	-	-		REF	-	-
	Other	0.81	(0.66, 0.99)	0.045		0.82	(0.37, 1.79)	0.614
Marital status	Married	REF	-	-		REF	-	-
	Widowed	1.02	(0.79, 1.32)	0.873		1.48	(0.77, 2.86)	0.239
	Divorced / separated	0.92	(0.74, 1.13)	0.420		0.72	(0.28, 1.85)	0.498
	Never married	1.02	(0.88, 1.18)	0.816		1.18	(0.63, 2.19)	0.605
Education	University degree	REF	-	-		REF	-	-
	College diploma	0.97	(0.87, 1.10)	0.671		0.60	(0.33, 1.08)	0.091
	Completed high school	0.93	(0.80, 1.10)	0.403		0.60	(0.33, 1.10)	0.101
	Did not graduate high school	0.83	(0.64, 1.07)	0.153		0.33	(0.15, 0.76)	0.009
Employment	Employed / student / disability / homemaker	REF	-	-		REF	-	-
	Unemployed	0.70	(0.36, 1.35)	0.284		1.53	(0.31, 7.52)	0.600
	Retired	1.05	(0.90, 1.24)	0.537		1.40	(0.63, 3.10)	0.413
	Other	1.03	(0.71, 1.49)	0.890		2.54	(0.80, 8.07)	0.115
Rural	No	REF	-	-		REF	-	-
	Yes	1.02	(0.91, 1.15)	0.742		1.65	(0.94, 2.89)	0.081
AUDIT score	0 (abstain)	REF	-	-		REF	-	-
	1 to 7 (low risk)	1.04	(0.91, 1.18)	0.586		0.97	(0.54, 1.73)	0.914
	> 7 (moderate to high risk)	0.95	(0.76, 1.19)	0.676		0.96	(0.41, 2.24)	0.917
Cannabis use frequency	Never	REF	-	-		REF	-	-
	Monthly to weekly	1.01	(0.86, 1.18)	0.918		0.65	(0.27, 1.55)	0.328
	Daily	1.04	(0.82, 1.33)	0.733		0.64	(0.21, 1.90)	0.419
Non-medical prescription opioid use	No	REF	-	-		REF	-	-
	Yes	1.45	(1.19, 1.78)	< 0.001		0.18	(0.03, 0.97)	0.047
Cocaine use (lifetime)	No	REF	-	-		REF	-	-
	Yes	0.94	(0.75, 1.17)	0.575		3.04	(1.37, 6.72)	0.006
K6 score	0 to 7	REF	-	-		REF	-	-
	≥8	0.98	(0.81, 1.17)	0.798		0.71	(0.29, 1.70)	0.438

Table 5 shows the results of the randomized trial of the order of questions on the willingness to provide biological samples and PHI. Unweighted proportions and chi-squared tests of demographics by randomized group are listed in Table S3 in the supplement. No significant differences in demographic variables were observed between groups. Individuals who were asked first to permit direct health insurance database linkage were 0.52 times less likely to be willing to provide a saliva sample than if they were asked first to provide a saliva sample (95% CI: 0.32, 0.82). In contrast, if individuals were asked first to provide a saliva sample, they were 0.84 times less likely to agree to probabilistic health insurance database linkage than if they were asked first for permission for probabilistic linkage (95% CI: 0.73–0.97). The order of questions did not affect willingness to provide a blood sample or direct health insurance database linkage.

**Table 5:** Prevalence ratios of obtaining positive answers to biological sample collection and data requests by question order, 2019 CAMH Monitor study

Data requested	First question asked	Prevalence ratio		
		Point estimate	(95% Confidence Interval)	*p*-value
Blood sample				
	Blood sample	REF	-	-
	Probabilistic health insurance database linkage	1.01	(0.59, 1.75)	0.959
	Direct health insurance database linkage	1.14	(0.70, 1.87)	0.594
Saliva sample				
	Saliva sample	REF	-	-
	Probabilistic health insurance database linkage	0.90	(0.64, 1.26)	0.550
	Direct health insurance database linkage	0.52	(0.32, 0.83)	0.006
Probabilistic health insurance database linkage				
	Probabilistic health insurance database linkage	REF	-	-
	Saliva sample	0.84	(0.73, 0.97)	0.022
	Blood sample	0.91	(0.80, 1.03)	0.132
Direct health insurance database linkage				
	Direct health insurance database linkage	REF	-	-
	Saliva sample	1.46	(0.80, 2.66)	0.218
	Blood sample	1.26	(0.68, 2.36)	0.466

Asking participants for their OHIP number before asking them to provide a saliva sample resulted in far fewer participants responding positively to both requests compared to participants who were first asked to provide a saliva sample and then asked to provide their OHIP number (PR: 0.31, 95% CI: 0.12, 0.80). For all other combinations, there were no indications that question order affected the probability of receiving a positive response to either of the biological sample or PHI questions (see Table S4 in the supplement).

## Discussion

This study demonstrates that requests for blood samples, saliva samples, and direct linkage with health insurance database requests are problematic, as only a small proportion of respondents were willing to provide such information. This reluctance in sharing biological samples could be attributed to a lack of understanding about the (future) use of blood and saliva samples for research, a lack of trust and privacy concerns, and wariness or superstitions resulting from cultural views on providing biological samples [34–36]. Probabilistic linkage requests were more promising, with the majority of respondents willing to have their PHI probabilistically linked. Studies conducted in the past have found that survey respondents are generally willing to have their PHI probabilistically linked; however, data linkage consent rates tend to vary from study to study [37, 38]. Further research is required to expose factors which contribute to a lack of willingness to provide biological samples and direct data linkage.

When these associations were examined by the various demographic and substance use/abuse variables, it was found that gender, employment status, rurality, AUDIT score, and frequency of cannabis use in the past 12 months did not significantly affect willingness to provide PHI and biological samples. It was noted that 75 + year old individuals had an increased willingness to provide blood samples compared to respondents who were 30 to 39 years of age. The factors informing this latter finding are unclear, as the literature on the willingness of older adults to participate in research is contradictory. Specifically, many studies indicate that willingness to participate decreases with age, whereas others characterize older adults as curious and interested in research [39–41]. Therefore, future research is required to investigate the factors that contribute to older adults’ willingness to provide blood samples, specifically. Increased willingness to provide blood samples among people with high K6 scores could be due to the general phenomena of sick people being motivated by their individual need to participate in research that could benefit them [42]. Additionally, research is needed to confirm the finding that divorced people were signficantly less likely to provide a blood sample (compared to married people) as this finding is at odds with the finding that divorced people were not signficantly less likely to provide a saliva sample or to provide permission to have their PHI probabilistically or directly linked.

Fewer respondents who identified with a racial/ethnic category other than White were willing to provide saliva samples and have their PHI probabilistically linked. It has been noted previously that ethnic minorities have higher rates of non-consent for linkage [43]. This systematic bias could be attributable to ethnic minorities’ mistrust of health systems, and of how their PHI will be used [44]. Similarly, individuals who did not complete high school or just completed high school had a lower prevalence of willingness to provide blood samples and direct linkage with health insurance databases compared to those who had completed university degrees. Individuals of lower educational achievement have previously shown a decreased willingness to provide PHI [45]. It is assumed that an individual’s education level may affect their literacy and understanding, such that those individuals who are more highly educated may recognize and understand the benefits of research more than their less educated counterparts [46, 47].

With regards to substance use, individuals who had used cocaine in their lifetime showed a higher willingness to provide blood samples and direct linkage with health insurance databases. Interestingly, individuals who had engaged in NMPOU use in the past 12 months were less likely to be willing to allow direct linkage, but were more willing to allow probabilistic linkage. NMPOU users may be worried about the potential ramifications on their safe supply of opioids by admitting to NMPOU, as the requested PHI is used in the prescription process [48, 49]. In other words, such users may fear that consenting to direct linkage could lead to the blocking of their source of medical opioids. As a person’s access to cocaine is not impacted by providing PHI, this may explain why cocaine users showed higher willingness to provide blood samples and PHI.

Question order effects were observed such that individuals who were asked first to agree to direct linkage of PHI were less likely to be willing to provide a saliva sample, and individuals who were asked first for a saliva sample were less likely to agree to probabilistic linkage. Furthermore, asking participants for their OHIP number before asking them to provide a saliva sample, rather than the reverse, resulted in a decreased likelihood of responding positively to both requests. Varied evidence of question order effects have been observed when participants were asked for consent to multiple data linkages; however, there appears to be no literature probing the presence of question order effects when asking for both PHI data linkage and biological samples [50].

### Limitations

Small sample sizes may have resulted in low statistical power for some analyses. Sampling bias may have contributed to observed low response rates and absolute proportions of respondents willing to provide biological samples and PHI for data linkage. Future research with larger sample sizes is needed to confirm these effects. It must also be noted that the questions posed regarding willingness were hypothetical. It is possible that response rates may differ if participants are actually asked to provide biological samples and PHI for linkage. Imbalances within race categories, specifically the overrepresentation of participants who identified as ‘White’, poses generalizability issues. Finally, cocaine use and opioid use were posed as binary questions. The frequency of drug use could reveal differential distributions in willingness to provide biological samples and PHI for data linkage, as observed in the cannabis item.

## Public health relevance

Irrespective of these limitations, the findings retain immense public health relevance. Difficulty in collecting biological samples is often viewed as “the rate-limiting step” for clinical research and personalized medicine [51]. Identifying willingness to provide biological samples and levels of agreement to data linkage can prompt future qualitative studies to investigate methods to increase consent rates. Furthermore, the question order effects observed in this study have important implications for population health survey design. Specifically, depending on the type of PHI requested first, it may not be advisable to subsequently ask for biological data, or vice versa. Increasing willingness for probabilistic and data linkage can improve data completeness and quality, which in turn can be used to answer pertinent clinical and public health questions [52, 53].

Though question order effects enable researchers to strategically design surveys in order to increase respondents’ willingness for linkage, there is potential for misuse and manipulation if there is a lack of ethical consideration of the limits of its utility. When intentionally using question order effects in data linkage studies, it is essential to consult and seek approval from committees concerning the potential for question ordering to unduly persuade individuals to provide permission for probabilistic and direct data linkage to health records. Alternative methods exist to improve participant willingness to permit probabilistic and direct data linkage to health records. These methods include the framing of how data will be utilized and shared after linkage, developing trust (e.g., data security and privacy), and prioritizing informed consent, respondent understanding, and transparency [54, 55].

Finally, low willingness of minority groups to provide PHI for linkage and biological samples can prevent these populations from experiencing the benefits of health research [56]. The inevitable underrepresentation of minorities in future cohorts will result in any new, pertinent health information and treatments being biased towards those of ‘White’ racial identities. As per a mandate from the WHO, quantifying these health inequities is of utmost public health importance, as the advancement of health research without an equitable distribution of benefits is of limited value [57]. Thus, efforts to increase trust in the process of research, including the collection of PHI and biological data among under-represented racial groups, should be a priority.

## Conclusion

Our study suggests that although there is opposition to providing biological samples and PHI for data linkage, these attitudes tend to vary by different demographic, mental health, and substance use characteristics. Furthermore, willingness to provide such information can differ based on the order of requests.

Data linkage of PHI and biological samples presents invaluable information for health research. By linking PHI and biological samples to survey responses, researchers can disaggregate epidemiological trends by specific cohorts and their associated characteristics. Subsequent studies investigating the prevalence of willingness to provide biological samples and PHI need to be undertaken to verify these results and to aid in the formation of evidence-based strategies to increase consent rates.

## Electronic supplementary material

Below is the link to the electronic supplementary material.

Supplementary Material 1

## Data Availability

The data that support the findings of this study are available from the Centre for Addiction and Mental Health, but restrictions apply to the availability of these data, which were used under license for the current study, and so these data are not publicly available. However, data are available from the authors upon reasonable request and with permission of the Centre for Addiction and Mental Health.

## Abbreviations

AUDIT: Alcohol Use Disorders Identification Test
CI: Confidence interval
ICES: Institute for Clinical and Evaluative Sciences
K6: Kessler Psychological Distress Scale
NESARC-III: National Epidemiologic Survey on Alcohol and Related Conditions-III
NMPOU: Non-medical prescription opioid use
OHIP: Ontario Health Insurance Plan
PHI: Personal health information
PR: Prevalence ratios
WHO: World Health Organization
