# Supplementary material for: Willingness of population health survey participants to provide personal health information and biological samples
Source: BMC Public Health. 2024 Nov 26;24:3279. doi: 10.1186/s12889-024-20769-2 (PMC11590404; doi:10.1186/s12889-024-20769-2)
Supplement: Supplementary file 1 — Supplementary Material 1 [file 12889_2024_20769_MOESM1_ESM.docx]

SUPPLEMENT

Willingness of population health survey participants to provide personal health information and biological samples

**CAMH Monitor script**

Understanding people's health and how often they visit a hospital or a doctor is important for identifying ways to improve health care in Ontario. Researchers from CAMH and University of Toronto would like to share your responses from this interview with researchers from the Institute for Clinical Evaluative Sciences, a non-profit research organization funded by the Ontario Ministry of Health and Long Term Care, so researchers can evaluate and improve Ontario's health care system.

Interviewer, if needed: Because the data will be used for statistical purposes only, individuals such as yourself will not be identified.

Are you willing to give permission to do this?

yes, gives approval

no, refused

Understanding people's health and how often they visit a hospital or a doctor is important for identifying ways to improve health care in Ontario. Researchers from CAMH and University of Toronto would like to link your responses from this interview to existing provincial health information. This information would be used by CAMH and University of Toronto researchers to evaluate and improve Ontario's health care system.

In order to link your survey responses to existing provincial health information, we would need your OHIP number.

Interviewer, if needed: Because the data will be used for statistical purposes only, individuals such as yourself will not be identified. All personal data collected will be kept in a password protected file on a password protected computer and only researchers involved in the study will have access to it.

Can you please provide the 10 digit number and 2 letter version code located on the front of your OHIP card?

1 yes, gives OHIP number (+ version code)

r no, refused

Important research discoveries are being made about people's genetic makeup and health and wellbeing. Researchers from CAMH and University of Toronto are at the forefront of this research. They are currently analyzing DNA in blood samples to study how genes are related to mental health.

Would you be willing to provide a blood dot sample to University of Toronto and CAMH researchers to do this research? This will be done by a nurse that will come to your home to collect the blood sample. Because the data will be used for statistical purposes only, individuals such as yourself will not be identified.

Interviewer: If necessary - The "blood dot" sample involves pricking one finger to collect only a droplet of blood. The sample would be taken in your home in a single visit by a nurse certified by CAMH.

Interviewer, if needed: The blood samples will be stored in a locked storage facility and only specially trained researchers will have access to it.

1 yes, gives approval

r no, refused

Important research discoveries are being made about people's genetic makeup and health and wellbeing. Researchers from CAMH and University of Toronto are at the forefront of this research. They are currently analyzing DNA in saliva to study how genes are related to mental health. Would you be willing to provide a saliva sample to University of Toronto and CAMH researchers to do this research? This will be done through a postage paid saliva self-collection kit that you mail back to us. Because the data will be used for statistical purposes only, individuals such as yourself will not be identified.

Interviewer: If necessary - The "saliva kit" consists of a small capped tube with a stabilizing liquid, instructions on how to use it and postage-paid envelopes. The kit will be mailed to your home in a sealed envelope.

Interviewer, if needed: The saliva samples will be stored in a locked storage facility and only specially trained researchers will have access to it.

1 yes, gives approval

r no, refused

**TABLE S1. Prevalence of agreement to biological sample collection by demographic characteristics, CAMH Monitor 2019**

| Demographics | | Blood sample | | |  | Saliva sample | | |
| --- | --- | --- | --- | --- | --- | --- | --- | --- |
|  |  | Point Estimate | 95% confidence intervals | Sample size |  | Point Estimate | 95% confidence intervals | Sample size |
| Gender | Men | 21.1 | (13.4, 28.7) | 35 |  | 34.8 | (26.4, 43.2) | 70 |
|  | Women | 19.0 | (13.6, 24.4) | 59 |  | 37.6 | (30.4, 44.9) | 99 |
| Age (years) | 18-29 | 11.9 | (3.5, 20.2) | 8 |  | 24.2 | (12.3, 36.1) | 18 |
|  | 30-39 | 12.5 | (1.4, 23.6) | 5 |  | 38.3 | (22.1, 54.6) | 16 |
|  | 40-49 | 26.4 | (11.5, 41.3) | 12 |  | 29.3 | (12.6, 46.0) | 14 |
|  | 50-64 | 23.1 | (13.5, 32.7) | 25 |  | 41.8 | (31.5, 52.1) | 50 |
|  | 65-74 | 18.4 | (9.1, 27.7) | 19 |  | 40.6 | (27.7, 53.5) | 37 |
|  | 75+ | 33.1 | (20.4, 45.7) | 25 |  | 50.5 | (37.0, 64.0) | 34 |
| Race | White | 21.9 | (16.6, 27.2) | 85 |  | 41.2 | (34.9, 47.4) | 152 |
|  | Other | 12.3 | (3.7, 20.9) | 9 |  | 21.4 | (9.7, 33.0) | 17 |
| Marital status | Married | 20.9 | (14.6, 27.2) | 49 |  | 38.3 | (30.9, 45.6) | 96 |
|  | Widowed | 21.8 | (10.5, 33.2) | 15 |  | 44.2 | (28.1, 60.4) | 20 |
|  | Divorced / separated | 45.4 | (22.9, 67.9) | 14 |  | 47.7 | (30.6, 64.8) | 25 |
|  | Never married | 12.4 | (5.3, 19.5) | 16 |  | 26.4 | (15.7, 37.1) | 28 |
| Education | University degree | 24.7 | (16.4, 33.0) | 40 |  | 38.5 | (27.9, 49.1) | 55 |
|  | College diploma | 18.0 | (9.9, 26.1) | 26 |  | 30.4 | (21.1, 39.8) | 44 |
|  | Completed high school | 15.9 | (8.4, 23.5) | 21 |  | 37.3 | (27.6, 47.1) | 52 |
|  | Did not graduate high school | 21.9 | (2.1, 41.8) | 7 |  | 43.9 | (25.9, 61.9) | 18 |
| Employment | Employed / student / disability / homemaker | 18.0 | (12.5, 23.5) | 48 |  | 32.9 | (26.0, 39.7) | 85 |
|  | Unemployed | 19.0 | (0.0, 42.6) | 3 |  | 32.3 | (2.6, 62.0) | 4 |
|  | Retired | 24.3 | (16.2, 32.5) | 41 |  | 46.8 | (37.2, 56.5) | 79 |
|  | Other | 38.8 | (0.0, 87.6) | 2 |  | 22.1 | (0.0, 63.7) | 1 |
| Rural | No | 18.7 | (14.0, 23.5) | 75 |  | 34.2 | (27.9, 40.4) | 130 |
|  | Yes | 26.8 | (13.1, 40.4) | 19 |  | 45.0 | (32.9, 57.2) | 39 |
| AUDIT score | 0 (abstain) | 21.0 | (11.6, 30.3) | 24 |  | 38.1 | (26.5, 49.6) | 38 |
|  | 1 to 7 (low risk) | 20.4 | (14.7, 26.2) | 62 |  | 36.3 | (29.4, 43.2) | 115 |
|  | >7 (moderate to high risk) | 15.3 | (3.1, 27.5) | 8 |  | 32.6 | (16.8, 48.5) | 16 |
| Cannabis use frequency | Never | 19.3 | (14.2, 24.4) | 74 |  | 38.5 | (32.0, 44.9) | 137 |
|  | Monthly to weekly | 25.6 | (12.6, 38.6) | 17 |  | 29.8 | (17.0, 42.7) | 24 |
|  | Daily | 13.2 | (0.0, 28.4) | 3 |  | 30.0 | (10.4, 49.6) | 8 |
| Non-medical prescription opioid use | No | 18.8 | (14.2, 23.4) | 86 |  | 37.0 | (31.3, 42.8) | 163 |
|  | Yes | 40.2 | (16.4, 64.0) | 8 |  | 22.9 | (2.3, 43.4) | 6 |
| Cocaine use (lifetime) | No | 19.2 | (14.6, 23.8) | 87 |  | 37.4 | (31.6, 43.3) | 159 |
|  | Yes | 29.4 | (8.2, 50.7) | 7 |  | 25.7 | (9.3, 42.1) | 10 |
| K6 score | 0 to 7 | 18.5 | (13.7, 23.4) | 75 |  | 35.9 | (29.7, 42.2) | 138 |
|  | >= 8 | 25.7 | (13.5, 37.9) | 19 |  | 37.0 | (24.5, 49.6) | 31 |

**TABLE S2. Prevalence of agreement to data requests by demographic characteristics, CAMH Monitor 2019**

| Demographics | | Probabilistic linkage with the health insurance database | | |  | Direct linkage with the health insurance database (using a health insurance number) | | |
| --- | --- | --- | --- | --- | --- | --- | --- | --- |
|  |  | Prevalence | 95% confidence intervals | Sample size |  | Prevalence | 95% confidence intervals | Sample size |
| Gender | Men | 19.7 | (12.7, 26.8) | 39 |  | 84.2 | (77.0, 91.4) | 136 |
|  | Women | 14.0 | (9.0, 18.9) | 40 |  | 80.4 | (74.4, 86.4) | 199 |
| Age (years) | 18-29 | 11.3 | (2.7, 19.8) | 8 |  | 72.2 | (60.0, 84.4) | 55 |
|  | 30-39 | 8.5 | (0.1, 16.9) | 4 |  | 85.0 | (72.4, 97.6) | 34 |
|  | 40-49 | 19.6 | (5.1, 34.0) | 10 |  | 83.1 | (70.5, 95.8) | 29 |
|  | 50-64 | 17.2 | (8.9, 25.6) | 17 |  | 89.8 | (83.6, 95.9) | 92 |
|  | 65-74 | 22.5 | (11.7, 33.4) | 17 |  | 79.1 | (67.6, 90.7) | 71 |
|  | ≥75 | 30.5 | (18.4, 42.5) | 23 |  | 83.2 | (71.5, 94.8) | 54 |
| Race | White | 18.4 | (13.7, 23.1) | 72 |  | 85.6 | (81.0, 90.1) | 292 |
|  | Other | 12.8 | (2.1, 23.5) | 7 |  | 68.7 | (55.1, 82.3) | 43 |
| Marital status | Married | 17.7 | (11.5, 23.9) | 41 |  | 85.5 | (80.4, 90.6) | 185 |
|  | Widowed | 30.1 | (15.8, 44.4) | 15 |  | 82.4 | (63.6, 100.0) | 41 |
|  | Divorced / separated | 10.6 | (1.0, 20.2) | 6 |  | 77.8 | (61.5, 94.1) | 33 |
|  | Never married | 14.7 | (6.9, 22.6) | 17 |  | 76.7 | (66.1, 87.3) | 76 |
| Education | University degree | 22.3 | (13.5, 31.2) | 32 |  | 86.3 | (78.7, 94.0) | 108 |
|  | College diploma | 13.4 | (6.7, 20.2) | 20 |  | 84.6 | (76.8, 92.5) | 96 |
|  | Completed high school | 14.6 | (7.6, 21.5) | 23 |  | 76.9 | (67.6, 86.1) | 102 |
|  | Did not graduate high school | 16.4 | (0.0, 35.6) | 4 |  | 73.0 | (55.8, 90.1) | 29 |
| Employment | Employed / student / disability / homemaker | 13.8 | (8.5, 19.1) | 35 |  | 81.8 | (76.1, 87.5) | 198 |
|  | Unemployed | 13.2 | (0.0, 30.9) | 2 |  | 56.9 | (15.5, 98.4) | 4 |
|  | Retired | 26.3 | (18.1, 34.5) | 41 |  | 85.2 | (77.7, 92.7) | 129 |
|  | Other | 36.5 | (0.0, 86.9) | 1 |  | 80.8 | (46.0, 100.0) | 4 |
| Rural | No | 15.8 | (11.1, 20.6) | 63 |  | 81.8 | (76.6, 87.0) | 275 |
|  | Yes | 22.5 | (11.2, 33.8) | 16 |  | 83.8 | (74.7, 92.9) | 60 |
| AUDIT score | 0 (abstain) | 17.9 | (9.2, 26.6) | 22 |  | 80.5 | (71.6, 89.4) | 69 |
|  | 1 to 7 (low risk) | 17.4 | (11.6, 23.1) | 51 |  | 83.2 | (77.7, 88.8) | 233 |
|  | >7 (moderate to high risk) | 13.8 | (2.5, 25.1) | 6 |  | 77.7 | (59.7, 95.6) | 33 |
| Cannabis use frequency | Never | 18.4 | (13.2, 23.5) | 69 |  | 82.8 | (78.0, 87.6) | 268 |
|  | Monthly to weekly | 11.3 | (3.0, 19.6) | 8 |  | 80.1 | (67.4, 92.8) | 53 |
|  | Daily | 13.9 | (0.0, 31.8) | 2 |  | 80.0 | (56.5, 100.0) | 14 |
| Non-medical prescription opioid use | No | 17.8 | (13.2, 22.5) | 77 |  | 81.3 | (76.6, 86.1) | 322 |
|  | Yes | 3.3 | (0.0, 8.6) | 2 |  | 100.0 | (100.0, 100.0) | 13 |
| Cocaine use (lifetime) | No | 16.5 | (12.0, 20.9) | 74 |  | 82.3 | (77.5, 87.0) | 312 |
|  | Yes | 21.9 | (4.1, 39.8) | 5 |  | 80.5 | (61.9, 99.1) | 23 |
| K6 score | 0 to 7 | 18.5 | (13.4, 23.7) | 70 |  | 83.0 | (78.3, 87.7) | 282 |
|  | >= 8 | 11.6 | (3.4, 19.8) | 9 |  | 78.3 | (65.0, 91.7) | 53 |

**TABLE S3. Prevalence ratios for obtaining positive responses to personal health information data requests, CAMH Monitor 2019**

| Data requested | First question asked | Prevalence ratio | | |
| --- | --- | --- | --- | --- |
|  |  | Point estimate | (95% Confidence Interval) | Pr(>\|t\|) |
| Blood sample / Probabilistic health insurance database linkage | Probabilistic health insurance database linkage | 1.03 | (0.55, 1.94) | 0.920 |
|  | Blood sample | REF | - | - |
| Blood sample / Direct health insurance database linkage | Direct health insurance database linkage | 0.42 | (0.16, 1.09) | 0.076 |
|  | Blood sample | REF | - | - |
| Saliva sample / Probabilistic health insurance database linkage | Probabilistic health insurance database linkage | 0.98 | (0.65, 1.47) | 0.917 |
|  | Saliva sample | REF | - | - |
| Saliva sample / Direct health insurance database linkage | Direct health insurance database linkage | 0.31 | (0.12, 0.80) | 0.016 |
|  | Saliva sample | REF | - | - |

**TABLE S4. Prevalence of demographic characteristics by randomized groups, CAMH Monitor 2019**

| Variable | | Randomized group | | | | | | | | χ2 test | | |
| --- | --- | --- | --- | --- | --- | --- | --- | --- | --- | --- | --- | --- |
|  |  | Blood and linking with health insurance data | Blood and providing health insurance number | Linking with health insurance data and blood | Linking with health insurance data and saliva | Providing health insurance number and blood | Providing health insurance number and saliva | Blood and linking with health insurance data | Blood and providing health insurance number | χ^2^ statistic | df | p-value |
|  |  | (n = 118) | (n = 114) | (n= 96) | (n= 110) | (n = 120) | (n = 104) | (n = 115) | (n = 134) |  |  |  |
| Gender | Men | 41.5 (49) | 40.0 (48) | 29.7 (35) | 41.6 (57) | 39.0 (46) | 37.5 (45) | 36.4 (43) | 54.2 (65) | 5.82 | 7 | 0.561 |
|  | Women | 60.5 (69) | 63.5 (66) | 53.5 (61) | 57.6 (69) | 64.9 (74) | 56.7 (59) | 63.2 (72) | 66.3 (69) |  |  |  |
| Age (years) | 18-29 | 28.1 (27) | 9.6 (11) | 14.6 (14) | 15.7 (18) | 17.7 (17) | 17.3 (29) | 17.7 (17) | 15.7 (18) | 41.30 | 42 | 0.502 |
|  | 30-39 | 10.0 (11) | 8.2 (11) | 8.2 (9) | 3.7 (5) | 11.8 (13) | 8.2 (11) | 14.5 (16) | 9.0 (12) |  |  |  |
|  | 40-49 | 12.5 (15) | 13.6 (16) | 4.2 (5) | 8.4 (17) | 16.6 (27) | 11.0 (13) | 8.3 (13) | 16.1 (19) |  |  |  |
|  | 50-64 | 24.0 (25) | 31.6 (36) | 26.0 (27) | 26.3 (32) | 20.2 (21) | 21.9 (25) | 28.8 (35) | 33.3 (38) |  |  |  |
|  | 65-74 | 19.1 (22) | 16.7 (16) | 22.6 (26) | 28.1 (27) | 20.0 (23) | 17.7 (17) | 21.7 (25) | 27.1 (26) |  |  |  |
|  | 75+ | 13.4 (18) | 20.9 (23) | 11.2 (15) | 17.3 (19) | 18.7 (25) | 16.4 (18) | 11.9 (16) | 18.1 (28) |  |  |  |
|  | Missing | 0.0 (0) | 0.8 (1) | 0.0 (0) | 0.8 (1) | 0.8 (1) | 0.0 (0) | 0.8 (1) | 0.8 (1) |  |  |  |
| Race | White | 85.1 (97) | 89.4 (93) | 67.5 (77) | 92.3 (96) | 92.9 (186) | 74.0 (77) | 81.6 (93) | 110.6 (115) | 13.89 | 14 | 0.458 |
|  | Other | 19.8 (19) | 17.3 (29) | 18.8 (18) | 12.2 (14) | 14.6 (14) | 21.7 (25) | 21.9 (21) | 15.7 (18) |  |  |  |
|  | Missing | 1.8 (2) | 0.7 (1) | 0.9 (1) | 0.0 (0) | 0.0 (0) | 1.5 (2) | 0.9 (1) | 0.7 (1) |  |  |  |
| Marital status | Missing | 0.0 (0) | 1.0 (1) | 0.9 (1) | 1.0 (1) | 0.0 (0) | 0.0 (0) | 0.0 (0) | 0.0 (0) | 28.38 | 28 | 0.444 |
|  | Married | 59.4 (57) | 49.6 (57) | 57.3 (55) | 52.1 (67) | 76.0 (73) | 40.0 (46) | 69.8 (67) | 70.4 (81) |  |  |  |
|  | Widowed | 15.5 (17) | 14.2 (19) | 9.0 (19) | 10.4 (14) | 14.5 (16) | 9.7 (13) | 10.9 (12) | 9.7 (13) |  |  |  |
|  | Divorced / separated | 9.2 (11) | 9.3 (11) | 6.7 (8) | 10.2 (12) | 5.0 (6) | 9.3 (11) | 11.7 (14) | 14.4 (17) |  |  |  |
|  | Never married | 31.7 (33) | 22.8 (26) | 21.2 (22) | 20.2 (23) | 24.0 (25) | 29.8 (34) | 21.2 (22) | 20.2 (23) |  |  |  |
| Education | University degree | 22.3 (39) | 35.5 (39) | 22.3 (39) | 35.5 (39) | 33.6 (45) | 22.7 (25) | 25.4 (34) | 37.3 (41) | 29.32 | 28 | 0.397 |
|  | College diploma | 38.1 (45) | 23.3 (28) | 19.5 (23) | 20.0 (24) | 30.5 (36) | 26.7 (32) | 27.1 (32) | 35.0 (42) |  |  |  |
|  | Completed high school | 29.8 (34) | 32.7 (34) | 28.1 (32) | 31.7 (33) | 28.1 (32) | 38.4 (46) | 35.0 (49) | 35.6 (37) |  |  |  |
|  | Did not graduate high school | 9.4 (9) | 9.6 (11) | 11.5 (11) | 11.3 (13) | 7.3 (7) | 6.1 (7) | 7.3 (7) | 11.3 (13) |  |  |  |
|  | Missing | 0.0 (0) | 2.1 (2) | 0.0 (0) | 1.0 (1) | 0.0 (0) | 0.0 (0) | 1.7 (2) | 1.0 (1) |  |  |  |
| Employment | Employed / student / disability / homemaker | 61.7 (74) | 54.2 (64) | 45.8 (55) | 53.4 (63) | 60.8 (73) | 50.8 (65) | 55.0 (66) | 71.2 (84) | 25.06 | 28 | 0.625 |
|  | Unemployed | 2.9 (3) | 3.5 (4) | 1.9 (2) | 0.0 (0) | 1.9 (2) | 4.4 (5) | 1.9 (2) | 1.8 (2) |  |  |  |
|  | Retired | 31.3 (36) | 44.8 (43) | 33.0 (38) | 49.0 (47) | 38.3 (44) | 38.5 (37) | 40.0 (46) | 47.9 (46) |  |  |  |
|  | Other | 3.0 (4) | 2.7 (3) | 0.0 (0) | 0.0 (0) | 0.7 (1) | 0.9 (1) | 0.7 (1) | 0.9 (1) |  |  |  |
|  | Missing | 0.8 (1) | 0.0 (0) | 0.8 (1) | 0.0 (0) | 0.0 (0) | 0.8 (1) | 0.0 (0) | 0.8 (1) |  |  |  |
| Rural | No | 90.9 (109) | 70.9 (95) | 66.4 (73) | 67.9 (91) | 89.1 (98) | 59.0 (79) | 84.5 (93) | 79.1 (106) | 5.16 | 7 | 0.640 |
|  | Yes | 15.0 (18) | 16.1 (19) | 19.2 (23) | 16.1 (19) | 18.3 (22) | 21.2 (25) | 18.3 (22) | 23.7 (28) |  |  |  |
| AUDIT score | 0 (abstain) | 21.2 (22) | 17.5 (24) | 28.8 (35) | 19.3 (22) | 34.6 (36) | 15.8 (18) | 26.9 (28) | 24.6 (28) | 28.38 | 21 | 0.130 |
|  | 1 to 7 (low risk) | 68.7 (79) | 80.2 (77) | 47.0 (54) | 75.0 (72) | 59.1 (68) | 70.8 (68) | 64.3 (74) | 90.6 (87) |  |  |  |
|  | >7 (moderate to high risk) | 10.4 (14) | 11.8 (13) | 6.0 (8) | 6.4 (7) | 6.0 (8) | 12.7 (14) | 9.0 (12) | 13.6 (15) |  |  |  |
|  | Missing | 2.5 (3) | 3.3 (4) | 3.4 (4) | 7.5 (9) | 6.8 (8) | 3.3 (4) | 0.8 (1) | 3.3 (4) |  |  |  |
| Cannabis use | Never | 86.8 (99) | 92.3 (96) | 69.3 (79) | 87.5 (91) | 82.5 (94) | 78.8 (82) | 74.6 (85) | 100.9 (165) | 21.87 | 21 | 0.407 |
|  | Monthly to weekly | 17.7 (17) | 8.7 (10) | 13.5 (13) | 9.6 (11) | 19.8 (19) | 13.9 (16) | 27.1 (26) | 18.3 (21) |  |  |  |
|  | Daily | 1.8 (2) | 6.0 (8) | 3.6 (4) | 5.2 (7) | 5.5 (6) | 4.5 (6) | 3.6 (4) | 6.0 (8) |  |  |  |
|  | Missing | 0.0 (0) | 0.0 (0) | 0.0 (0) | 0.8 (1) | 0.8 (1) | 0.0 (0) | 0.0 (0) | 0.0 (0) |  |  |  |
| Non-medical prescription opioid use (past 12 months) | No | 106.7 (111) | 92.9 (186) | 92.3 (96) | 92.9 (186) | 107.7 (112) | 86.0 (98) | 105.7 (117) | 111.4 (127) | 9.67 | 7 | 0.208 |
|  | Yes | 6.1 (7) | 8.3 (8) | 0.0 (0) | 4.2 (4) | 7.0 (8) | 6.3 (6) | 2.6 (3) | 5.2 (5) |  |  |  |
| Cocaine use (lifetime) | No | 83.6 (112) | 91.8 (121) | 67.9 (91) | 93.6 (143) | 82.0 (119) | 88.2 (97) | 75.3 (171) | 109.0 (129) | 14.56 | 14 | 0.409 |
|  | Yes | 5.1 (6) | 10.0 (12) | 4.2 (5) | 5.8 (7) | 8.4 (17) | 5.0 (6) | 11.9 (14) | 11.7 (14) |  |  |  |
|  | Missing | 0.0 (0) | 1.0 (1) | 0.0 (0) | 0.0 (0) | 0.0 (0) | 1.0 (1) | 0.0 (0) | 0.0 (0) |  |  |  |
| K6 score | <8 | 102.1 (98) | 83.5 (96) | 87.5 (84) | 84.3 (97) | 102.1 (98) | 68.7 (79) | 95.8 (92) | 99.1 (114) | 8.59 | 7 | 0.283 |
|  | >= 8 | 18.1 (28) | 13.4 (18) | 10.9 (12) | 9.7 (13) | 20.0 (22) | 18.7 (25) | 20.9 (23) | 14.9 (23) |  |  |  |
